# Replication-Competent Recombinant Porcine Reproductive and Respiratory Syndrome (PRRS) Viruses Expressing Indicator Proteins and Antiviral Cytokines

**DOI:** 10.3390/v4010102

**Published:** 2012-01-18

**Authors:** Yongming Sang, Jishu Shi, Wenjing Sang, Raymond R. R. Rowland, Frank Blecha

**Affiliations:** 1 Department of Anatomy and Physiology, College of Veterinary Medicine, Kansas State University, Manhattan, KS 66506, USA; Email: ysang@vet.k-state.edu (Y.S.); jshi@vet.k-state.edu (J.S.); 2 Departmentof Diagnostic Medicine and Pathobiology, College of Veterinary Medicine, Kansas State University, Manhattan, KS 66506, USA; Email: wenjing@k-state.edu (W.S.); browland@vet.k-state.edu (R.R.R.R.)

**Keywords:** porcine arterivirus, virus cDNA infectious clone, indicator proteins, type I interferon, host factors, tumor susceptibility gene 101

## Abstract

Porcine reproductive and respiratory syndrome virus (PRRSV) can subvert early innate immunity, which leads to ineffective antimicrobial responses. Overcoming immune subversion is critical for developing vaccines and other measures to control this devastating swine virus. The overall goal of this work was to enhance innate and adaptive immunity following vaccination through the expression of interferon (IFN) genes by the PRRSV genome. We have constructed a series of recombinant PRRS viruses using an infectious PRRSV cDNA clone (pCMV-P129). Coding regions of exogenous genes, which included *Renilla* luciferase (Rluc), green and red fluorescent proteins (GFP and DsRed, respectively) and several interferons (IFNs), were constructed and expressed through a unique subgenomic mRNA placed between ORF1b and ORF2 of the PRRSV infectious clone. The constructs, which expressed Rluc, GFP, DsRed, efficiently produced progeny viruses and mimicked the parental virus in both MARC-145 cells and porcine macrophages. In contrast, replication of IFN-expressing viruses was attenuated, similar to the level of replication observed after the addition of exogenous IFN. Furthermore, the IFN expressing viruses inhibited the replication of a second PRRS virus co-transfected or co-infected. Inhibition by the different IFN subtypes corresponded to their anti-PRRSV activity, *i.e.*, IFNω5 ° IFNα1 > IFN-β > IFNδ3. In summary, the indicator-expressing viruses provided an efficient means for real-time monitoring of viral replication thus allowing high‑throughput elucidation of the role of host factors in PRRSV infection. This was shown when they were used to clearly demonstrate the involvement of tumor susceptibility gene 101 (TSG101) in the early stage of PRRSV infection. In addition, replication‑competent IFN-expressing viruses may be good candidates for development of modified live virus (MLV) vaccines, which are capable of reversing subverted innate immune responses and may induce more effective adaptive immunity against PRRSV infection.

## 1. Introduction

More than 20 years after initial reports [1,2,3], porcine reproductive and respiratory syndrome virus (PRRSV) continues to be a global swine industry problem with losses in the U.S. approaching $6 billion over the last decade [4]. Belonging to the arteriviridae family in the order nidovirales, PRRSV is an enveloped RNA virus containing a single positive-strand RNA genome. The 15 kb viral RNA genome consists of seven open reading frames (ORF1-7). ORF1 comprises about 80% of the genome and encodes proteins with protease, replicase and regulatory functions. The smaller overlapping ORF2-7 encode five minor (GP2a, GP3, GP4, 5a and E proteins) and three major (GP5, M and N proteins) structural proteins [5,6,7]. Several studies have shown that PRRSV possesses the capacity to subvert early innate immune responses in pigs by suppressing the production of antiviral cytokines [8,9,10,11,12,13,14,15], which also contributes to ineffective B- and T-cell responses [16,17,18,19]. Superimposed on this suppressive activity is a high viral mutation rate, which has made the development of vaccines challenging [20]. Modified live vaccines (MLV) used for control of PRRSV in the U.S. are based on only two virus isolates [20,21]. Although MLV protect against some homologous field strains, their efficacy is not satisfactory due to failure to protect against infections of heterologous strains, as well as the potential risk for reversion to virulence [20,21]. To develop successful vaccines against PRRSV infections, particularly those by heterologous strains, it is necessary to develop novel vector systems and to extensively categorize host factors critical in the virus-host interaction. 

Several viral vectors, including those based on pseudorabies virus, poxvirus, adenovirus and transmissible gastroenteritis coronavirus (TGEV) have been used to express PRRSV structural proteins [22] or host immune factors [23,24,25] for developing anti-PRRSV immunity. For example, humoral immunity against PRRSV GP5 protein was detected in pigs immunized with fowlpoxvirus-coexpressing PRRSV GP5/GP3 and porcine IL-18 [23], and in mice immunized with adenovirus-expressing GP5/GP3 fused with swine granulocyte-macrophage colony stimulating factor (GM-CSF) [25]. In this context, we and others have proposed to use PRRSV infectious cDNA clones [26,27] or virus replicons [28] as vectors for the expression of immune effectors that potentiate innate and adaptive immunity against a broad range of PRRSV isolates. Here we show that PRRSV infectious clones are effective vector systems to express exogenous antigens and host immune effectors. Specifically, we have constructed serial replication-competent viruses from a PRRSV infectious clone-based vector expressing indicator proteins and porcine type I interferons (IFNs). The indicator protein-expressing PRRS viruses efficiently produce progeny viruses and provide an efficient means for real-time monitoring of viral replication, thus allowing high-throughput elucidation of the role of host factors in PRRSV infection. In addition, the replication of some IFN-incorporated viruses is associated with the expression of active IFN peptides, which are capable of counteracting the subverted innate immune response and with potential to induce more effective adaptive immunity against PRRSV infection.

## 2. Results and Discussion

### 2.1. Producing Replication-Competent PRRSV Coexpressing Indicator Proteins

**Figure 1:**
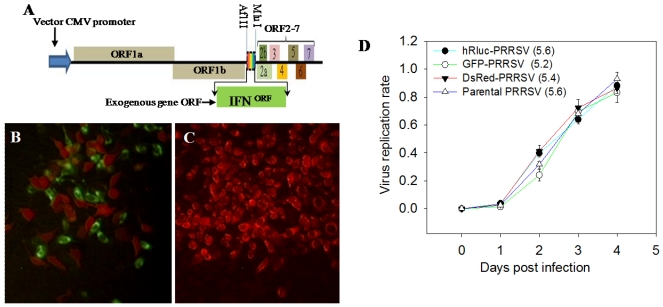
Infectious Porcine reproductive and respiratory syndrome virus (PRRSV) cDNA clone as a vector for bioengineered expression of indicator proteins. (**A**) Schematic of an infectious PRRSV cDNA clone, pCMV-P129-GFP, as an expression cassette [29]. (**B**) Co-infection of GFP- and DsRed-viruses (2nd passage of the rescued viruses, P2) in MARC-145 cells. Viruses were demonstrated by fluorescence of the indicator proteins. (**C**) Infection of porcine myeloid-derived dendritic cells with the *Renilla* luciferase (Rluc) (P6). Viruses were demonstrated by immunostaining PRRSV N protein indirectly labeled with TRITC. (**D**) Propagation dynamics of engineered viruses, values in parentheses are titers (log^TCID50/mL^) of P2 viruses.

To investigate the potential of infectious PRRSV cDNA clones as a platform for gene manipulation [29,30], we first engineered an established infectious clone to express several indicator proteins including *Renilla* luciferase (Rluc), and green and red fluorescent proteins (GFP and DsRed, respectively). Coding regions of the indicator proteins were constructed in the junction regions surrounding ORF1b/ORF2a through introduced restrictive digestion sites as described [29,30] (Figure 1A). The construct was designed to express the recombinant protein gene through the creation of an additional subgenomic mRNA. Plasmids of selected authentic clones were transfected into MARC-145 cells for the production of progeny viruses. As shown in Figure 1, infectious clones efficiently produced progeny viruses with successful expression of indictor proteins (Figure 1B). Replication rates of these engineered viruses were similar to their parental virus as judged by monitoring ratios of virus-positive cells; and they produced comparable infectious virons shown by similar viral titers (Figure 1C). Further experiments with GFP-PRRSV have shown their similar infectivity as a parental strain in porcine alveolar macrophages (PAMs) as well as monocyte-derived dendritic cells (mDCs) (Figure 1B and data not shown). The indicator-expressing viruses provide an efficient means for real-time monitoring of viral replication. Whereas GFP- and DsRed-PRRSV facilitated detection of fluorescent proteins after 16 h, the Rluc-PRRSV was useful for measuring Rluc activity from 5–20 h. 

These viruses allowed us to efficiently elucidate the role of some host factors in PRRSV infection. Several infectious cDNA clones have been generated from field PRRSV strains [31,32,33,34,35,36,37], which provide efficient means for molecular manipulation of PRRSV genome and evaluation of molecular evolution of this RNA virus with a high mutation rate. The vector cassette used in this study was generated from an infectious clone of North American PRRSV strain P129 with two unique restriction sites and a copy of the transcription regulatory sequence of ORF6 (TRS6) inserted between ORFs 1b and 2a [26,27,29,30]. In addition to GFP, which was introduced in PRRSV infectious clones in several previous studies [26,27,29,31], two other indicator proteins of DsRed and Rluc were introduced in the PRRSV cDNA clone in this study. The construction of DsRed and particularly Rluc into the PRRSV cDNA clone was intended to produce laboratory viruses that mimic their parental PRRSV with similar replication kinetics but are easily detected and quantified during the early phase of virus infection/replication. As shown in both MARC-145 cells and PAMs, all GFP, DsRed and Rluc expressing progeny viruses had similar replication kinetics as their parental PRRSV. For detection, the red fluorescence of DsRed labeling not only provided a counterstaining choice but also was more sensitive for microscopic observation in real-time rather than traditional immunostaining procedures in fixed cells (Rural Technologies, Brookings, SD, USA). In contrast to GFP and DsRed viruses, which were generally detectable after 12 h in infected cells using microscopy, the Rluc PRRSV in conjunction with an EnduRen™ *in vivo* substrate (Promega, Madison, WI) facilitated real-time detection of the virus replication as early as 5 h post infection in cells. This Rluc expressing PRRSV thus provides an efficient means for genome-wide examination of host factors involved in the early stages of virus infection [38,39]. However, our attempts to clone the firefly luciferase gene (~1.6 kb) into the same PRRSV cDNA vector were unsuccessful, suggesting that the PRRSV clone vector has a limited capacity for incorporation of exogenous genes at about 2 kb. 

### 2.2. Indicator Proteins Expressing PRRS Viruses Reveal the Involvement of Tumor Susceptibility Gene 101 (TSG101) in the Early Stage of PRRSV Infection

**Figure 2:**
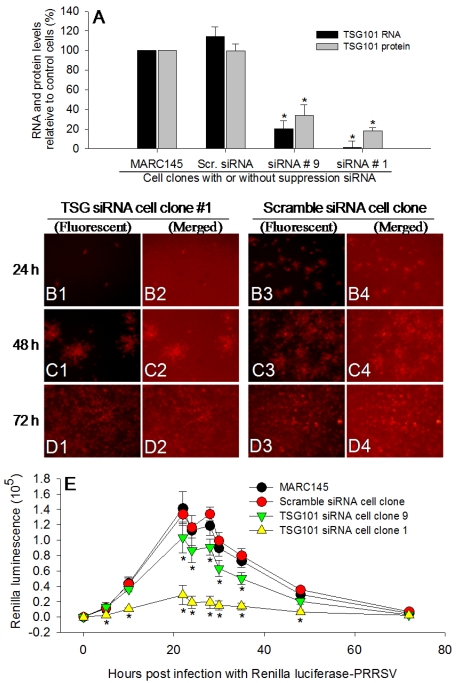
Indicator protein-expressing PRRS viruses are effective tools for deciphering the role of host factors in PRRSV infection. (**A**) Producing cell clones of MARC-145 cells with the suppression of a cellular protein of tumor susceptibility gene 101 (TSG101). (**B1–D4**) Retarded replication of PRRSV in TSG101-suppressed cells (B1–D2) as shown with DsRed-PRRSV in comparison to control cells (B3–D4). Merged: merged with fluorescent and bright field. (**E**) Real-time monitoring retarded replication of PRRSV in TSG101‑suppressed cells with the luciferase activity of the recombinant Rluc-PRRSV. The significant suppression of PRRSV replication in early phase but not latter phase of infection is clearly shown with the real-time measure using the indicator protein-expressing viruses, which is critical for detecting the window of a host factor involved in PRRSV infection. * *p* < 0.05, n = 3, to normal or scramble shRNA transfected cells.

TSG101 is a housekeeping protein and has been implicated in a number of cellular functions, including mitotic spindle formation, genome stability and endosomal sorting [40]. Essential in endosomal sorting, TSG101 interacts directly with ubiquitinated proteins and internalizes them into the multivesicular body pathway for degradation. During replication, viruses require a retrograde movement from the cell interior to the outer membrane [40]. A number of reports have shown that TSG101 is involved in the virus fusion/budding process from the cellular membrane. These viruses include human immunodeficiency virus (HIV) and hepatitis C virus (HCV) [40,41,42]. Recently, a tentative TSG101-targeting peptide, FGI-104, was shown to have a broad-spectrum ability to inhibit infections by several viruses including HIV, HCV and PRRSV [43]. However, there is no direct mechanistic evidence that has demonstrated the involvement of TSG101 in the control of PRRSV replication. To study this possibility, we produced MARC-145 cell lines with targeted suppression of endogenous TSG101 expression. MARC-145 cells were transfected with a pGFP-V-RS vector expressing a 29 nt shRNA (OriGene, Rockville, MD, USA) against a conserved region of TSG101. Two puromycin-resistant colonies showing significant suppression of TSG101 at both RNA and protein levels, were selected. As shown in Figure 2A, cells from colony 1 had less than 10% endogenous expression of *tsg101* RNA, and those from colony 9 about 20% of endogenous expression of *tsg101* RNA, as well as 70–80% reduction in TSG101 protein. We then compared PRRSV replication in these TSG101-suppressed cells with control MARC-145 cells or cells transfected with scrambled shRNA constructs. Using either GFP- or DsRed-expressing PRRSV, the retarded replication of viruses in TSG101-suppressed cells was demonstrated between 12 and 48 h after infection (Figure 2B,C). However, by 72 h, viral replication among TSG101-suppressed and control cells was not significantly different (Figure 2D). The return of PRRSV replication in TSG101-suppressed cells might result from virus-stimulated expression of TSG101 and/or incomplete suppression. The earlier and more quantitative comparison of PRRSV replication among TSG101-suppressed and control cells was conducted with the Rluc-expressing PRRSV. Significantly retarded viral replication was detected as early as 5 h post Rluc-PRRSV infection with the *in vivo* Rluc substrate. The difference in PRRSV replication was quantitatively correlated to the TSG101 levels in different group of cells (Figure 2A and 2E), and could be monitored until 20 h post infection when the substrate was limited. Notably, the replication retardation of indicator protein-expressing viruses in TSG101-silent cells was mostly due to TSG101 suppression because these bioengineered viruses have similar replication kinetics as their parental viruses in normal cells (Figure 1). Using the kinetics (*i.e.*, 12–20 h post infection) defined by the indicator protein-expressing viruses, we reproducibly detected retarded replication rates of wild-type PRRS viruses in TSG101 suppressed cells (data no shown). To further test the role of TSG101 in PRRSV infection in porcine cells or pigs, we have characterized the porcine *TSG101* cDNA sequence (GenBank^TM^ accession numbers JN882576). The full-length porcine *TSG101* cDNA is 1580 bp encoding a precursor protein of 391 residues. TSG101 genes are conserved with most mammalian homologs sharing >94% identity at both RNA and protein levels. The shRNA template sequence we used for loss-of-function studies in MARC-145 cells is identical among human, monkey and porcine TSG101 cDNA. Transient transformation of the shRNA into porcine mDCs similarly suppressed GFP, DsRed- and Rluc-expressing PRRSV until 48 h post infection (data not shown). 

### 2.3. Replication-Competent PRRSV Coexpressing Type I Interferons

**Figure 3:**
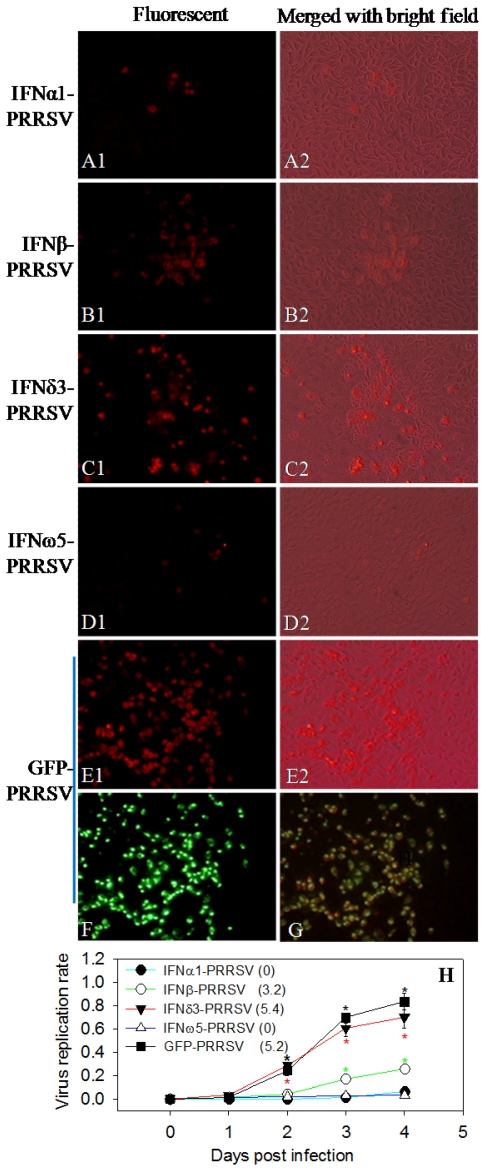
Infectious PRRSV cDNA clone as a vector for bioengineered expression of immune effectors. (**A–E**) MARC-145 cells were transfected with equal amounts of indicated infectious constructs, and immunostained for PRRSV at 4 d post-transfection with antibody against the PRRSV N protein indirectly labeled with TRITC; (**F**) GFP‑PRRSV immunostained as in E1 but shown here as green fluorescence of GFP; (**G**) Merged (E1) and (F) to show specificity of the immunostaining procedure; (**H**) Propagation rate of IFN-engineered viruses, values in parentheses are titers (log^TCID50/mL^) of P2 viruses. * *p* < 0.05, n = 3, to IFNα1-PRRSV.

**Figure 4:**
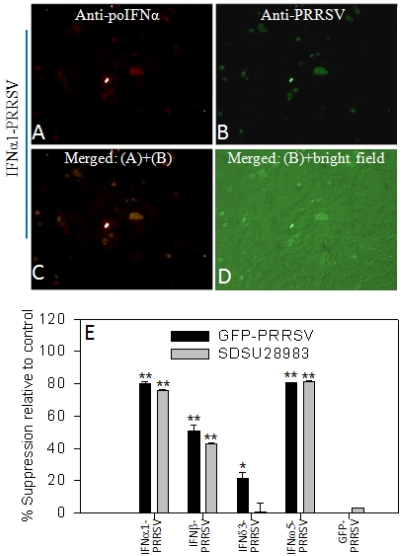
Interferon (IFN)-engineered viruses express active IFN peptides and induce anti-PRRSV activity in MARC-145 cells. (**A–D**) Counterstained PRRSV and IFN-α1 in IFN-α1-PRRSV transfected cells at 4 d post transfection; (**E**) IFN-engineered PRRSV showed suppression of co-transfected (GFP-PRRSV) or co-infected (SDSU28983, infected at 3 d post transfection) viruses. Total virus-infected cells were examined at 5 d post transfection to calculate suppression rate in comparison to controls. ** *p* < 0.01, * *p* < 0.05, n = 3, to cells only transfected with GFP-PRRSV.

To determine the potential of host factors as a means of counteracting viral immunomodulating activity and stimulating effective antiviral immunity [44], we bioengineered the infectious clones to express a variety of type I IFNs, which were selected because of their well-documented role in PRRSV infections and activity in mediation of antiviral immunity [9,15,45,46,47]. In addition, the suppression of type I IFN expression by PRRSV infection has been well documented [15]. Shown in Figure 3 are data from the infectious clone expressing four type I IFNs (IFNα1, IFNβ, IFN*δ*3 and IFNω5). At 4 d post transfection with equivalent cDNA clones, virus-positive cells were detected in cells transfected with all four infectious clones but with different densities. Where IFN*δ*3-PRRSV propagated similarly as the control GFP-virus, the replication rates of IFN-β-, IFNα1- and IFNω5-viruses were attenuated by approximately 70% (β-type) or 90% (α1- & ω5-types) (Figure 3A–H). Attenuation of IFN‑expressing viruses could be caused by viral genome alteration or more likely by the replication‑associated expression of active IFN peptides, which was consistent with the anti-PRRSV activity of these IFN subtypes [15] (Figure 3C). In contrast, IFN*δ*3- and GFP-type viruses replicated well. To confirm these findings, we counterstained IFNα1-virus infected cells with antibodies against porcine IFNα (R&D, Minneapolis, MN, USA) and PRRSV nucleocapsid (N) protein (Figure 4A–D). Co‑localization of IFNα- and PRRSV-labeling (Figure 4C) indicated that IFN polypeptides were expressed by the engineered viruses during infection. Furthermore, the IFN expressing viruses inhibited the replication of a second PRRS virus co-transfected or co-infected; and again, the intensity of the inhibition was consistent with the anti-PRRSV activity of these IFN subtypes, *i.e.*, IFNω5°IFNα1>IFN-β>IFN*δ*3 (Figure 4E). However, because the expression of IFN inhibits virus replication [22,44], engineered viruses with the most active IFN subtypes against PRRSV may not be good candidates for a modified live virus (MLV) vaccine. This limitation would prevent the preparation of a high titer virus as a vaccine. Several measures may be used to overcome these limitations, such as incorporating IFN subtypes that do not inhibit replication of a MLV strain but may up-regulate B- and T-cell responses [44], or controlled expression/activation of the incorporated IFN peptides within certain temporal windows or cell types. Pigs have at least 35 functional type I IFN genes with diverse antiviral or immunoregulatory activity [47], which provides several candidates for balancing antiviral and immunoregulatory activity to optimize the replication-competent recombinant viruses. Type I IFNs mediate antiviral responses through induction of IFN stimulated genes, such as *MxA* and *RNase L*. Direct incorporation of some ISGs (or their functional domains) into the PRRSV infectious clone provides an attractive alternative given that PRRSV-specific ISGs have been identified [15]. In addition, incorporating an exogenous gene tag (a compliance marker) at the vector backbone of a MLV will also allow differentiation of vaccinated from non-vaccinated animals [27]. 

As for the stability of the bioengineered viruses, we passed viruses through MARC-145 cells and PAMs for 5–6 generations. Authentic progeny viruses were rescued after three generations, but viruses with a titer higher than 10^3^ were only rescued with the indicator protein expressing group and the one expressing IFN*δ*3. DsRed- and IFN*δ*3-expressing viruses remained stable for 6 generations (Data not shown). 

## 3. Experimental Section

*Viruses and cells:* All virus and animal procedures were approved by the Kansas State University Biosafety and Institutional Animal Care and Use committees. The wild type strains of PRRSV used here were two North American PRRSV strains: NVSL97-7895 and SDSU28983 [26,27]. The expression cassette was created by insertion of two unique restriction sites and a copy of the transcription regulatory sequence of ORF6 (TRS6) between ORFs 1b and 2a in the infectious cDNA clone of pCMV-P129 (Figure 1A) [29,30] (gift from Dr. Jay G. Calvert, Pfizer Animal Health, Kalamazoo, MI, USA). All bioengineered viruses originated from the backbone of pCMV-P129 with incorporation of an exogenous gene between the two cloning sites. All PRRSV strains were propagated in a simian kidney cell line (MARC-145) or porcine alveolar macrophages (PAMs). MARC-145 cells were maintained in minimum essential medium (MEM) with 8% fetal bovine serum (FBS) and 1× penicillin/streptomycin and fungizone (Invitrogen, Grand Island, NY, USA) PAMs were obtained from lungs of 4- to 6-week-old pigs by lung lavage with PBS and cryopreserved in liquid nitrogen until use. In use, PAMs were plated in RPMI medium with 10% FBS plus 1X penicillin/streptomycin and fungizone. After 2 d, cells were infected with the virus [47]. Monocyte-origin dendritic cells (mDCs) were induced from porcine peripheral blood mononuclear cells (PBMCs) and infected with PRRS viruses as described in Loving *et al.* [48]. 

*Production and titration of recombinant PRRS viruses with expression of exogenous genes:* In brief, two restriction enzyme digestion sites (Afl II and Mlu I) were introduced into 5'- and 3'-ends of the coding regions, which were amplified from authentic cDNA clones using a high-fidelity PCR [15,47]. The cloning primers used for this study are listed in the Supplemental Table 1. The amplified coding regions were purified and cloned into the expression cassette. MARC-145 cells were transfected with authentic plasmids purified from *E. coli* clones for examination of the production of progeny viruses and expression of indicator proteins or IFNs. Transfection of MARC-145 in 24-well plates was performed with 2 μg of plasmid DNA using Lipofectamine™ 2000 (Invitrogen). Subsequent virus yields were measured by end-point titration of culture media on MARC-145 cells and PAMs. Serial 10-fold dilutions of virus were placed in 96-well tissue culture plates containing confluent MARC-145 cells or PAMs. Cells were fixed in 4% formaldehyde in PBS and the virus was detected by staining for the presence of nucleocapsid antigen using a mAb (SDOW-17, Rural Technologies, Brookings, SD, USA) labeled with TRITC-conjugated secondary antibodies. In addition, the recombinant viruses could be distinctly detected by the expression of fluorescent proteins. Results were reported as log^TCID50/mL^ [15,47]. The replication of Rluc-expressing PRRSV was monitored with the addition of an *in vivo Renilla* luciferase substrate (Promega, Madison, WI, USA) at 60 μM in cell culture medium and measured the luminescence after 2 h [49]. 

*Determination of growth kinetics and stability of the recombinant viruses in cells:* Culture supernatants from cells transfected with infectious clones were harvested at 5 d post-transfection and designated ‘passage (P) 1’. The P1 virus was used to inoculate fresh MARC-145 cells to collect P2 then P3 at an interval of 4–5 d between successive passages. Each passage virus was titrated, aliquoted and stored at −80 °C until use. Growth curves of the rescued viruses were evaluated by inoculating MARC-145 cells with P3 viruses at a MOI of 0.1. Aliquots of the supernatants of infected cells were collected at points with 10 h intervals until 100 h and the virus was titrated by determining log^TCID50/mL^ to monitor growth kinetics [26]. To determine the stability of the recombinant virus, the rescued P1 virus was passaged on MARC-145 and PAM cells until P6. Expression of indicator proteins and IFN along with virus replication was detected by RT-PCR, western blotting and immunofluorescence as described above [15,47]. 

*Producing MARC-145 cell lines with shRNA-mediated silencing of the Tsg101 gene:* Three μg of pGFP-V-RS vector expressing a scrambled shRNA or a 29 nt shRNA (OriGene, Rockville, MD, USA) against a conservative region between human and simian TSG101 cDNA, was used to transfect MARC-145 cells growing in 6-well culture plates. Transfected cells were selected with 0.8 µg/mL of puromycin (Invitrogen) to obtain individual colonies. Approximately 30 puromycin-resistant colonies were picked and two of them showing significant suppression of TSG101 at both RNA and protein levels were subcultured for loss-of-function studies of the role of TSG101 in PRRSV infection. Primers used for RT-PCR detection of *tsg101* were generated against consensus sequence of monkey, human and porcine *tsg101* cDNAs (GenBank^TM^ accession numbers, NM_001195481, NM_006292 and JN882576, respectively), which allowed us to detect both monkey and porcine *tsg101* with the same pair of primers. The primers were 5′-ATACCCTCCCAATCCCAGTGGTTA-3′ (sense) and 5′‑ATCCATYTCCTCCTTCATCCGCCA-3′ (antisense, Y = C or T). Anti-TSG101 monoclonal antibody was purchased from Sigma-Aldrich (St. Louis, MO, USA). The involvement of TSG101 in PRRSV infection was evaluated by comparing the replication kinetics of PRRS viruses between cells with or without TSG101-suppression [47]. 

*Data analyses:* Virus titrations were done with at least three repeats to report log^TCID50/mL^. Relative gene-expression data of real-time PCR was normalized against C_t_ values of the housekeeping gene (GAPDH) and the relative expression index (2^−ΔΔCt^) was determined in comparison to the base levels of control samples. Growth curves were generated with Sigmaplot 11.0 (Systat, San Jose, CA, USA) and the densitometry analysis of images were done by using AlphaEase FC Software (Alpha Inotech, Santa Clara, CA, USA) as described [47,50].

## 4. Conclusions

The PRRSV cDNA infectious clone, pCMV-129, has vector capacity to express exogenous genes of less than 2 kb, which allows reconstruction of the virus for bioengineering manipulation [29,30]. 

Recombinant PRRS viruses expressing several indicator proteins have replication and infection dynamics similar to the parental strain in both MARC-145 and porcine cells. Therefore, they may be used to decipher the role of host factors in PRRSV infection [38,39]. Using several indicator protein‑expressing viruses, in particular the Rluc-expressing PRRSV, we showed that TSG101 was significantly involved in PRRSV infection at the early phase of the infection.

Recombinant PRRS viruses expressing antiviral cytokines produce active cytokines in the infected cells and alter the replication of co-infected PRRSV. These constructs may be candidates for modified live virus vaccines, which could ameliorate subverted innate immune responses and potentially enhance adaptive immunity against PRRSV infection.

## Supplementary Files

Supplementary File 1: PDF-Document (PDF, 103 KB)
