# Supplementary material for: Replication-Competent Recombinant Porcine Reproductive and Respiratory Syndrome (PRRS) Viruses Expressing Indicator Proteins and Antiviral Cytokines
Source: Viruses. 2012 Jan 18;4(1):102–16. doi: 10.3390/v4010102 (PMC3280517; doi:10.3390/v4010102)
Supplement: Supplementary File 1: — PDF-Document (PDF, 103 KB) [file viruses-04-00102-s001.pdf]

Supplemental Table 1. Cloning and PCR primers.

Gene	Primer Name and Sequence (5' to 3')*		Product Size (bp)
Renilla luciferase (*hRluc*)	Sense[C]	GCTA*CTTAAG*GCCACCATGGCTTCCAAGGTGTA	935
	Antisense[C]	ATCGC*ACGCGT*TTACTGCTCGTTCTTCAGC	
DsRed	Sense[C]	GCTA*CTTAAG*GCCACCATGGCCTCCTCCGA	684
	Antisense[C]	ATCGC*ACGCGT*CTACAGGAACAGGTGGTGGC	
poIFN-α1	Sense[C]	GCTA*CTTAAG*CAATGGCCCCAACCTCAG	
	Antisense[C]	ATCGC*ACGCGT*TGTCACTCCTTCYTCCTGA	574
	Sense	GGCTCTGGTGCATGAGATGC	
	Antisense	CAGCCAGGATGGAGTCCTCC	197
poIFN-β	Sense[C]	GCTA*CTTAAG*CAATGGCTAACAAGTGCATCCT	
	Antisense[C]	ATCGC*ACGCGT*TCAGTTCCGGAGGTAATCTGT	563
	Sense	ATGTCAGAAGCTCCTGGGACAGTT	
	Antisense	AGGTCATCCATCTGCCCATCAAGT	246
poIFN-δ3	Sense[C]	GCTA*CTTAAG*CAATGGCTCAGATTTACTTGGT	
	Antisense[C]	ATCGC*ACGCGT*TGAGGATTCTTAGGTAAGGGA	512
	Sense	AGAACTTGTCTGCTGTCCATT	209
	Antisense	TTTGGAGAAGACACCGGA	
poIFN-ω5	Sense[C]	GCTA*CTTAAG*CCATGGCCTTCGTGCTCTCTCTA	
	Antisense[C]	ATCGC*ACGCGT*TCAAGGTGACCCCAGGTST	573
	Sense	TCATGCTCTCTCTACTGACAGC	300
	Antisense	TGGAGCTTGTCCAGGAGGA	
Tsg101	Sense	ATACCCTCCCAATCCCAGTGGTTA	211
	Antisense	ATCCATYTCCTCCTTCATCCGCCA (Y = C or T)	
GAPDH	Sense	TGGYATCGTGGAAGGRCTCAT (R = A or G)	
	Antisense	RTGGGWGTYGCTGTTGAAGTC (W = A or T)	370

* Note: [C]Primers used for cloning. The underlined are introduced sequences including the restrictive digestion sites (Afi II and Mlu I, respectively in sense and antisense primers in *italic*).
